# Extracellular Vesicles From KSHV-Infected Cells Stimulate Antiviral Immune Response Through Mitochondrial DNA

**DOI:** 10.3389/fimmu.2019.00876

**Published:** 2019-04-24

**Authors:** Hyungtaek Jeon, Jisu Lee, Suhyuk Lee, Su-Kyung Kang, Sang June Park, Seung-Min Yoo, Myung-Shin Lee

**Affiliations:** Department of Microbiology and Immunology, Eulji University School of Medicine, Daejeon, South Korea

**Keywords:** extracellular vesicles, interferon-stimulated gens, innate immunity, KSHV, virus, antiviral response

## Abstract

Kaposi's Sarcoma-associated herpesvirus (KSHV) is the etiologic agent of Kaposi's sarcoma, which is the most common cancer in acquired immune deficiency syndrome patients. KSHV contains a variety of immunoregulatory proteins. There have been many studies on the modulation of antiviral response by these immunoregulatory proteins of KSHV. However, the antiviral effects of extracellular vesicles (EVs) during *de novo* KSHV infection have not been investigated to our best knowledge. In this study, we showed that KSHV-infected cells induce interferon-stimulated genes (ISGs) response but not type I interferon in uninfected bystander cells using EVs. mRNA microarray analysis showed that ISGs and IRF-activating genes were prominently activated in EVs from KSHV-infected cells (KSHV EVs)-treated human endothelial cells, which were validated by RT-qPCR and western blot analysis. We also found that this response was not associated with cell death or apoptosis by virus infection. Mechanistically, the cGAS-STING pathway was linked with these KSHV EVs-mediated ISGs expressions, and mitochondrial DNA on the surface of KSHV EVs was one of the causative factors. Besides, KSHV EVs-treated cells showed lower infectivity for KSHV and viral replication activity than mock EVs-treated cells. Our results indicate that EVs from KSHV-infected cells could be an initiating factor for the innate immune response against viral infection, which may be critical to understanding the microenvironment of virus-infected cells.

## Introduction

Cells release vesicles of varying sizes both via the endosomal pathway and by budding from the plasma membrane. These vesicles are referred to by various names, such as exosomes, microvesicles, microparticles, and apoptotic bodies, collectively termed extracellular vesicles (EVs) (1). EVs are a heterogeneous collection of membrane-bound carriers with complex cargoes including proteins, lipids, and nucleic acids, which work as crucial players in intercellular communication (2).

In many aspects, EVs resemble viruses, especially an enveloped virus (3). Their size and structure share similar features. Both are surrounded by a lipid membrane that also contains cell membrane proteins. EVs carry genetic material, which can change the functions of the recipient cells. Apparently, unlike viruses, EVs do not cause infection and replication. However, increasing evidence indicates that EVs from virus-infected cells affect immune response during viral infection. Dreux et al. reported that EVs released from Hepatitis C virus-infected cells can induce interferon (IFN)-α release from uninfected plasmacytoid dendritic cells due to the viral RNA present within the EVs (4).

Type I IFN and interferon-stimulated genes (ISGs) are indispensable for vertebrates to control viral infection (5, 6). Induction of type I IFN gene expression is tightly regulated. Generally, primary *de novo* viral infection and reactivation from latency elicit a host antiviral immune response. However, Kaposi's sarcoma-associated herpesvirus (KSHV), the etiologic agent of Kaposi's sarcoma, has multiple mechanisms to block type I IFN response (7–9). Especially, various tegument proteins in the virion work on antagonizing type I IFN response from the viral entry stage. Indeed, a previous study showed that KSHV induced little or very weak antiviral response during *de novo* infection (10). However, the antiviral effect in bystander cells during *de novo* KSHV infection has not been investigated so far. In this study, we demonstrated that EVs from KSHV-infected cells (KSHV EVs) can induce ISGs but not type I IFNs in human endothelial cells through the cGAS-STING pathway. EVs were isolated prior to virion production from KSHV-infected cells, and cell death or apoptosis was not observed at this time. We showed that mitochondrial DNA on EVs was one of the associated-factors inducing ISG expression. These results are important to understand the microenvironment of virus-infected cells because currently, little is known regarding the fact that virus-infected cells induce antiviral responses in bystander cells using independent mechanisms from type I IFN. Furthermore, we found that *de novo* infection of KSHV and human herpes simplex virus type 1 are partially blocked in KSHV EVs-pretreated cells.

## Materials and Methods

### Cell Culture

Human umbilical vein endothelial cells (HUVECs) were purchased from Lonza (Allendale, NJ) and cultured in endothelial cell growth medium-2 (EGM-2; Lonza) bullet kit. Vero cells and lenti-X-293T cells were obtained from Korean Cell Line Bank (Seoul, South Korea) and Takara (Otsu, Japan), respectively. They were cultured in Dulbecco's modified Eagle's medium (DMEM; GE Healthcare, Little Chalfont, UK) supplemented with 10% fetal bovine serum (FBS; Wellgene, Seoul, South Korea) and 1% antibiotics (Lonza). The cells were maintained in a humidified atmosphere of 5% CO_2_ at 37°C according to the manufacturer's instructions. An absence of contamination of mycoplasma in all cultured cells was tested by mycoplasma detecting PCR every month as described previously (11).

### Virus Isolation and Infection

iSLK BAC16 cells harboring recombinant KSHV BAC16 were used as the source of the virus, as described previously (12). Infectious KSHV BAC16 virions from iSLK BAC16 cells were induced by treatment with doxycycline and sodium butyrate for 3 days. The culture supernatant was collected, filtered through a 0.22 μm filter, and centrifuged at 100,000 × g for 1 h. The pellet was resuspended in phosphate-buffered saline (PBS) and stored at −70°C as infectious viral particles. HUVECs were infected with KSHV according to methods used in a previous study (13).

### Affymetrix Whole Transcript Expression Array Analysis

The Affymetrix whole transcript expression array process was executed according to the manufacturer's protocol (GeneChip Whole Transcript PLUS reagent Kit, Thermo Scientific, Waltham, MA). cDNA was synthesized using the GeneChip WT (Whole Transcript) Amplification kit as described by the manufacturer. The sense cDNA was then fragmented and biotin-labeled with TdT (terminal deoxynucleotidyl transferase) using the GeneChip WT Terminal Labeling kit. Approximately 5.5 μg of labeled DNA target was hybridized to the Affymetrix GeneChip Human 2.0 ST Array at 45°C for 16 h. Hybridized arrays were washed and stained on a GeneChip Fluidics Station 450 and scanned on a GCS3000 Scanner (Affymetrix, Santa Clara, CA). Signal values were computed using the Affymetrix® GeneChip™ Command Console software. Six most significant biofunctions were identified using Ingenuity Pathways Analysis (Ingenuity Systems; www. Ingenuity.com). Data are based on transcripts differentially expressed in mock- or KSHV-infected cells-derived EV-treated HUVECs. The *P*-value indicates the likelihood that an association of the specific set of transcripts with the indicated process or pathway is the result of random chance. B-H *P*-value indicates *P*-values after Benjamini-Hochberg correction for multiple comparisons.

### Isolation of EVs by Differential Centrifugation

For EVs isolation, HUVECs were incubated in EGM-2 for 24 h. Cell supernatants were collected and centrifuged at 2,000 × g for 10 min to remove cells, followed by filtration through a 0.22-μm pore filter (Sartorius, Göttingen, Germany) to remove cell debris. The collected supernatant was then ultracentrifuged at 100,000 × g for 60 min, and the precipitate was resuspended with PBS.

### Nanoparticle Tracking Analysis

The size distribution and concentration of EVs were determined by NTA, using a ZetaView (Particle Metrix GmbH, Meerbusch, Germany). Preparations of EVs were diluted in PBS and passed through 0.22 μm filters before the analysis. The analysis parameters were as follows: max size 200, min size 20, brightness 20, sensitivity 75, and temperature 25°C.

### Quantitative Real-Time Reverse Transcription PCR (RT-qPCR)

Total RNA from cells was isolated by NucleoSpin RNA II as recommended by the manufacturer (MACHEREY-NAGEL Inc., Bethlehem, PA). Total RNA was reverse-transcribed to obtain the first-strand cDNA using the ReverTra Ace qPCR RT kit (TOYOBO CO, Osaka, Japan). Real-time PCR was performed using the SYBR^®^ FAST qPCR mix (Takara). The cycling conditions were as follows: 95°C for 30 s, 40 cycles of 95°C for 5 s, and 60°C for 10 s. The specificity of the amplified products was confirmed by analyzing the melting curves. All samples were tested in triplicates and normalized by β-actin or glyceraldehyde 3-phosphate dehydrogenase (GAPDH). The primers were synthesized by Genotech (Daejeon, South Korea) and their sequences are described in Table 1.

**Table 1:** List of primers used for PCR.

**Gene**	**Sense primer**	**Antisense primer**	**Amplicon**
IFI44L	ATC TCT GCC ATT TAT GTT GT	GTA GAA TGC TCA GGT GTA AT	153 bp
IFIT1	AAT AGA CTG TGA GGA AGG A	ATA GGC AGA GAT CGC ATA	139 bp
MX1	CAG GAC TAC GAG ATT GAG AT	GTT ATG CCA GGA AGG TCT A	170 bp
GAPDH	GGT ATC GTG GAA GGA CTC	GTA GAS GCA GGG ATG ATG	91 bp
β-actin	AGA GCT ACG AGC TGC CTG AC	AGC ACT GTG TTG GCG TAC AG	164 bp
IFN-α	AAT GCG GAC TCC ATC TTG	GGG CTG TAT TTC TTC TCT GT	130 bp
IFN-β	CAT TAC CTG AAG GCC AAG GA	CAG CAT CTG CTG GTT GAA GA	147 bp
tRNA-LEU(UUR)	CAC CCA AGA ACA GGG TTT GT	TGG CCA TGG GTA TGT TGT TA	107 bp
β2-microglobulin	TGC TGT CTC CAT GTT TGA TGT ATC T	TCT CTG CTC CCC ACC TCT AAG T	86 bp
IFIT1	TAG AAC AGG CAT CAT TAA CAA G	CTC CAG GGC TTC ATT CAT A	152 bp
IFIT3	GAC TGA ATC CTC TGA ATG C	CCT TAT TGA ATG GTG TCT GAT	78 bp
OAS1	TCA GTC AGC AGA AGA GAT AA	CAA TGA ACT TGT CCA GAG ATT	118 bp
cGAS	CCT GCT GTA ACA CTT CTT AT	TAG TCG TAG TTG CTT CCT AA	147 bp
NADH sub1	TTC TAA TCG CAA TGG CAT TCC T	AAG GGT TGT AGT AGC CCG TAG	146 bp
NADH sub5	TTC ATC CCT GTA GCA TTG TTC G	GTT GGA ATA GGT TGT TAG CGG TA	184 bp
IFI44	CGG TAA CAT TCG TGA TAG ATA	TCT GAG AGG AGA AGT ATT GA	152 bp
ISG15	GCA GAT CAC CCA GAA GAT	CCT TGT TAT TCC TCA CCA G	182 bp
KSHV ORF26	GGA GAT TGC CAC CGT TTA	ACT GCA TAA TTT GGA TGT AGT C	93 bp

### Western Blotting

Western blotting was performed as previously described (14) with minor modifications. Cellular proteins were isolated using 1 × RIPA buffer containing a protease inhibitor and a phosphatase inhibitor. The proteins were resolved by electrophoresis in a 10–15% SDS-polyacrylamide gel and transferred onto a nitrocellulose membrane (GE Healthcare). The membranes were blocked with 5% skim milk in Tris-buffered saline with 0.1% Tween 20. Rabbit monoclonal anti-STING (Cell Signaling Technology, Beverly, MA), Rabbit monoclonal anti-cGAS (Cell Signaling Technology), rabbit polyclonal anti-Rab27b (Bioss Antibodies Inc., Woburn. MA), mouse monoclonal anti-KSHV ORF65 (14), rabbit polyclonal anti-GAPDH (Cusabio, Houston, TX), rabbit polyclonal anti-calnexin (Bioss Antibodies Inc.), mouse monoclonal anti-HDAC1 (Santa Cruz Biotechnology, Santa Cruz, CA), mouse monoclonal anti-mtTFA (Santa Cruz Biotechnology), rabbit polyclonal anti-MX1 (Bioss Antibodies Inc.), rabbit polyclonal anti-IFIT1 (Bioss Antibodies Inc.), rabbit polyclonal anti-IFIT44L (Bioss Antibodies Inc.) and mouse monoclonal anti-β-actin antibodies (Sigma, St. Louis, MO) were used as primary antibodies. HRP-conjugated anti-rabbit or anti-mouse antibodies (Bethyl Laboratories Inc., Montgomery, TX) were used as secondary antibodies. The results were visualized using an ECL detection reagent (Bio-Rad, Hercules, CA).

### ELISA for Type I Interferon

Mock EVs or KSHV EVs added to HUVECs and incubate for 24 h, followed by isolating the culture supernatant. Type I interferon in the culture supernatant was analyzed by human interferon α and β ELISA kit (Cusabio) according to the manufacturer's instructions.

### Immunofluorescence Assay (IFA)

IFA was performed as previously described (14). A mouse monoclonal antibody to ORF65 was used for tracking of KSHV particles. Infection of KSHV was analyzed by detection of LANA using a rat monoclonal antibody to KSHV ORF73 (Abcam, Cambridge, MA).

### Tracking of EVs by Fluorescent Labeling

For fluorescent labeling of the EV membrane, Exo-Glow (System Bioscience, Palo Alto, CA), acridine orange nucleic acid-selective fluorescent dye was added to the purified EV according to the manufacturer's instructions. After EV membranes were fluorescently labeled, ultracentrifugation was performed at 100,000 × g for 60 min to remove the unlabeled dye. The labeled EVs were then added to HUVECs. After 4–8 h of incubation, the cells were gently washed with 1 × PBS and analyzed by flow cytometry or fluorescence microscopy.

### Flow Cytometry

Flow cytometry experiments were performed to assess the infectivity of KSHV, apoptosis, and tracking of labeled EVs. Cells suspended in 1% FBS/PBS were analyzed using a Guava easyCyte Flow Cytometer and the InCyte 3.1 software (Merck Millipore, Bedford, MA). For apoptosis assay, FITC Annexin V apoptosis detection kit (BD Bioscience, San Jose, CA) was used as recommended by the manufacturer's instruction.

### LDH Release Assay

Media from mock- or KSHV-infected HUVEC cells at 8 h of postinfection was isolated and centrifuged at 300 × g for 3 min. Cytotoxicity detection kit plus LDH (Roche, Mannheim, Germany) was used to measure lactate dehydrogenase (LDH) released from dead cells. The prepared culture media was added to the same volume of LDH reagent and incubated for 30 min in the dark. The absorbance was measured at 490 and 650 nm by a microplate reader (Molecular Devices, Silicon Valley, CA).

### Lentivirus Infections

Plasmids containing shRNAs for human Rab27b (TRCN0000293978 and TRCN0000294016, Sigma), STING (TRCN0000163029, TRCN0000163296, Sigma), cGAS (TRCN0000428336, TRCN0000128706, Sigma), or a scramble shRNA (#1864, Addgene, Cambridge, MA) were co-transfected with pPACKF1 packaging plasmid mix (System Bioscience) into Lenti-X-293T cells (Takara) using Lipofectamine 3000 transfection reagent (Thermo Scientific) as per the manufacturer's recommendations. HUVECs were infected with viral supernatants from 293T cells along with polybrene (5 μg/mL) for 24 h. After 10 days of selection with puromycin (0.5 μg/mL), the efficiency of knockdown was evaluated by western blotting.

### Analysis of Virion DNA of KSHV and Mitochondrial DNA (mtDNA)

The supernatants of KSHV-infected HUVECs were collected and centrifuged at 100,000 × g for 1 h. For detect virion DNA, the pellet was resuspended in 1 × DNase buffer and treated by RQ1 RNase-free DNase I (Promega, Madison, WI) at 37°C for 1 h. DNA was extracted from DNAase-treated virion or EVs using the QIAamp DNA blood mini kit (Qiagen, Hilden, Germany) according to the manufacturer's recommendations. Real-time PCR analysis was carried out using the SYBR^®^ FAST qPCR mix (Takara) with primers in Table 1. KSHV ORF26 and NADH sub1/5 was amplified to analyze virion DNA and mtDNA, respectively. The cycling conditions were as follows: 95°C for 30 s, 40 cycles of 95°C for 5 s, and 60°C for 10 s.

### Cell Viability Assay

Cell viability was measured by the WST-1 cell proliferation reagent (Roche) according to the manufacturer's protocol. Briefly, WST-1 reagent was added into cells on 96-well culture plate (1:10) and incubated for 90 min in a humidified atmosphere of 5% CO_2_ at 37°C. Absorbance at 450 nm was measured with the reference wavelength set at 650 nm.

### *In vitro* Antiviral Assay and Plaque Formation Assay

HUVECs were pretreated with or without 2-fold serial dilution of IFN-α starting from 1,000 to 1.8 U/mL for 24 h. HSV-1 at a multiplicity of infection (MOI) of 0.1–64 was added to the medium containing the cells using opti-MEM (Thermo Scientific) for 1 h at 37°C. Viral supernatant was then removed, and the cells were refreshed with complete medium. The medium was removed 48 h of post infection and cells were fixed with 10% formaldehyde solution for 20 min at room temperature. After fixation, cells were visualized with 0.4% crystal violet. The excessive dye was then removed by immersing the plate in PBS. Each treatment was performed in duplicate. For plaque formation assay to measure the MOI of HSV-1, different dilutions of supernatant from virus-infected cells were used to infect Vero cells in opti-MEM for 1 h, followed by overlaying 2% FBS in DMEM containing 1% agarose (Bio-Rad) to immobilize the virus. After 24 h, cells were fixed and visualized with crystal violet, and the plaques were enumerated.

### Statistical Analysis

Results are shown as the mean ± standard deviation. The two-tailed Student's *t*-test was used to assess the statistically significant difference between groups. Statistical significance at *P* < 0.05 and < 0.01 is indicated by ^*^ and ^**^, respectively.

## Results

### EVs From KSHV-Infected Cells Stimulate the Expression of ISG-Related Transcripts

**Figure 1:**
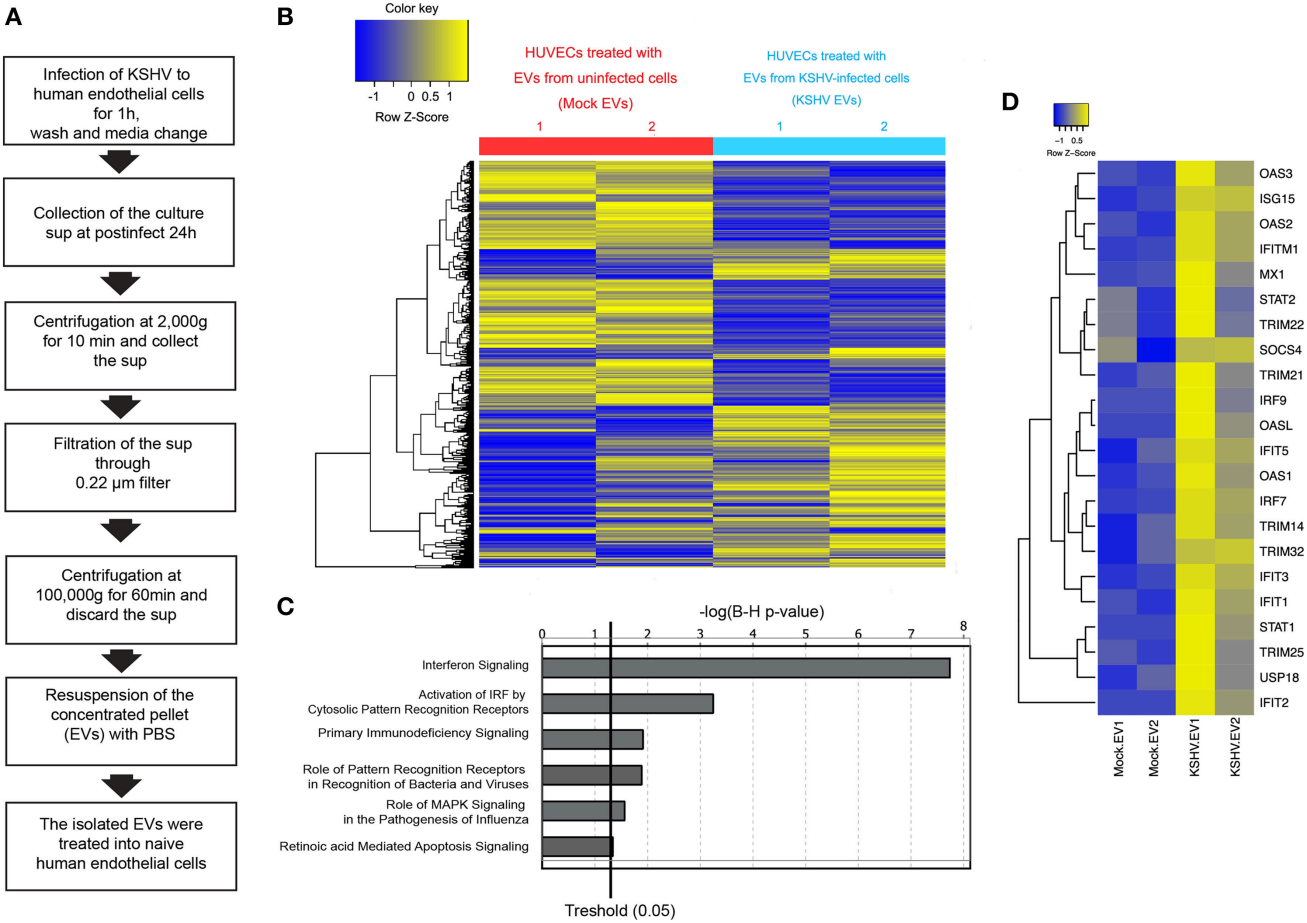
Microarray analysis for mRNA expression in human endothelial cells treated with EVs from mock- and KSHV-infected cells. **(A)** Schematic experimental processes of extracellular vesicle (EV) isolation from Kaposi's sarcoma-associated herpesvirus (KSHV)-infected human umbilical cord vein endothelial cells (HUVECs). Isolated EVs were treated to uninfected HUVECs, followed by analyzing for mRNA expression by microarray. **(B)** Hierarchical clustering analysis of mRNA levels in HUVECs treated with mock- vs. KSHV-infected cells-derived EVs (mock EVs vs. KSHV EVs). **(C)** Altered cell function and signal pathways in KSHV EVs-treated HUVECs as assessed by microarray analysis. **(D)** Heatmap for Interferon Stimulating Genes (ISGs) based on transcripts differentially expressed in mock- or KSHV-infected cells-derived EVs-treated HUVECs.

In the previous study, we isolated EVs from KSHV-infected human endothelial cells at 24 h of postinfection and characterized them (15). A schematic isolation process for EVs is presented as Figure 1A. Previously, EVs were analyzed by western blotting, nanoparticle tracking analysis, and electron microscopy. EVs-related proteins including CD81, CD63, and HSP70 were detected in EVs by western blot. We confirmed that these EVs were not contaminated with KSHV virions. In this study, we tried to investigate the influence of KSHV EVs on uninfected bystander cells. The isolated EVs from mock-infected (mock EVs) or KSHV-infected cells (KSHV EVs) were treated with naïve HUVECs for 24 h. Using a microarray, the differential expression of transcripts was analyzed with two sets of RNA samples independently prepared from EV-treated cells (Figure 1B). Gene expression profiling of KSHV EV-treated HUVECs revealed an enrichment of ISGs and antiviral signaling factors (Figures 1C,D). We observed increased expression of ISGs with direct antiviral activity (IFIT1, IFIT3, IFITM1, MX1, and OAS1) and positive regulators (cGAS, IRF4, IRF9, Stat1, and Stat2) reinforcing the antiviral response.

**Figure 2:**
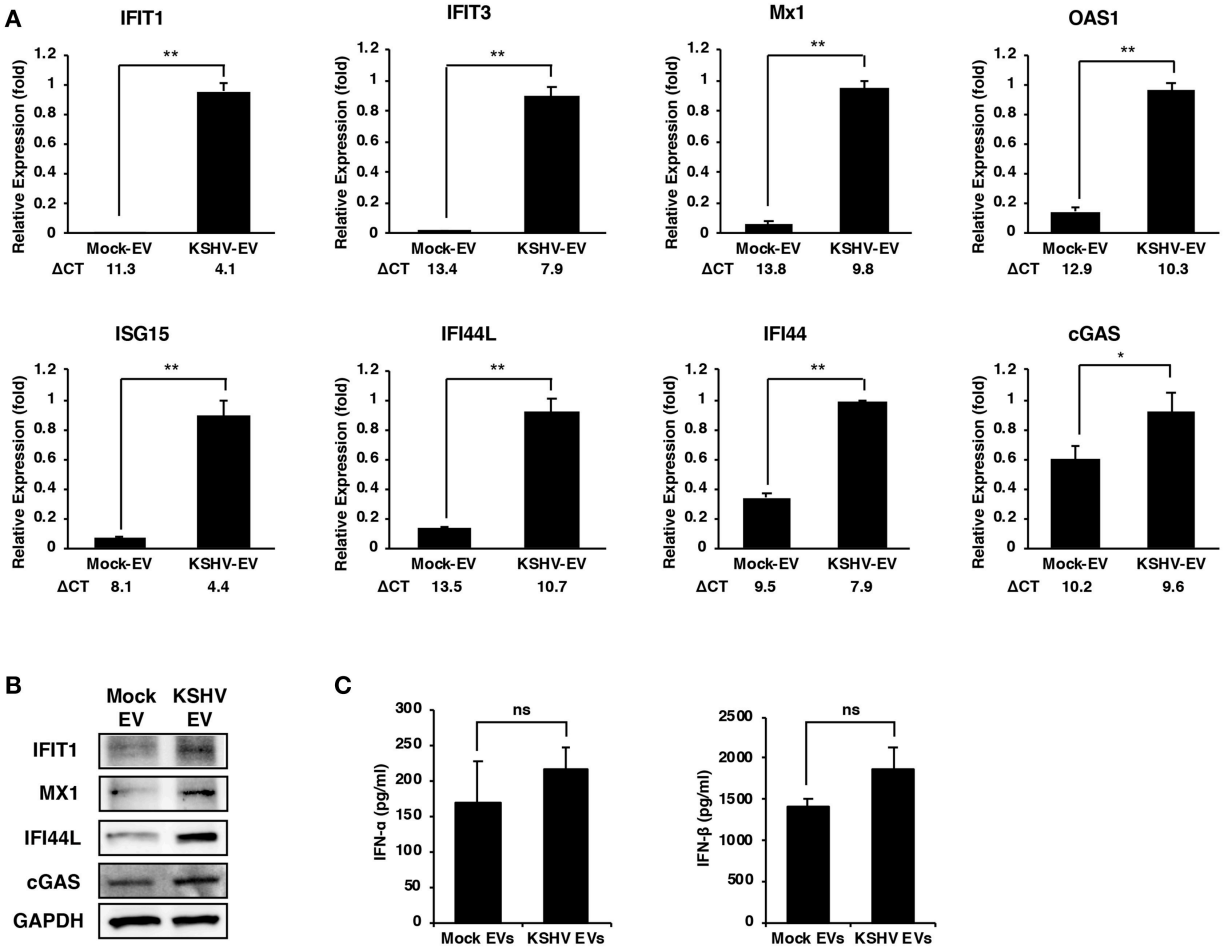
Increased expression of ISGs in human endothelial cells by EVs from KSHV-infected cells. **(A,B)** mRNA and protein expression of the indicated ISGs in mock- or KSHV EVs-treated HUVECs were analyzed by RT-qPCR **(A)** and western blotting **(B)**, respectively. ΔCT indicated the normalized CT value of ISGs with reference gene, β-actin. The grouping of blots cropped from different gels and full-length blots are included in a Supplementary Figure 4. Data are shown as the mean ± SD, *n* = 6, ^*^p < 0.05, ^**^p < 0.01. **(C)** Analysis for IFN-α and IFN-β in the supernatant from mock- or KSHV EVs-treated HUVECs by ELISA. Data are shown as the mean ± SD, *n* = 4, ns: not significant.

To validate the microarray results, the mRNA expression of ISGs in KSHV EVs-treated HUVECs were analyzed by RT-qPCR analysis (Figure 2A). Although there were some variations in differences between mock- and KSHV EVs-treated cells, the eight ISGs that were analyzed showed significant differences. We also validated the protein expression of IFIT1, MX1, IFI44L, and cGAS (Figure 2B). As ISGs have known to be induced by type I interferons (IFNs), we analyzed the expression of type I IFNs by KSHV EVs. After HUVECs were treated with mock EVs or KSHV EVs for 24 h, IFN-α and IFN-β in their supernatant were analyzed by ELISA (Figure 2C). A significant increase of type I IFNs was not observed in KSHV EVs-treated HUVECs compared to mock EVs-treated cells. A previous study showed that *de novo* KSHV infection suppressed the type I IFNs response by tegument proteins, ORF45, in KSHV (10). Interestingly, ISGs were highly upregulated in KSHV-infected cells at 24 h of postinfection (Supplementary Figure 1) in our results. These results suggest that KSHV EVs- or KSHV-infection-mediated ISG response might have an independent mechanism from type I IFN response of human endothelial cells.

### Stimulation of ISG Expression by KSHV EVs Is Not Associated With a Virus or a Product From Cell Death

**Figure 3:**
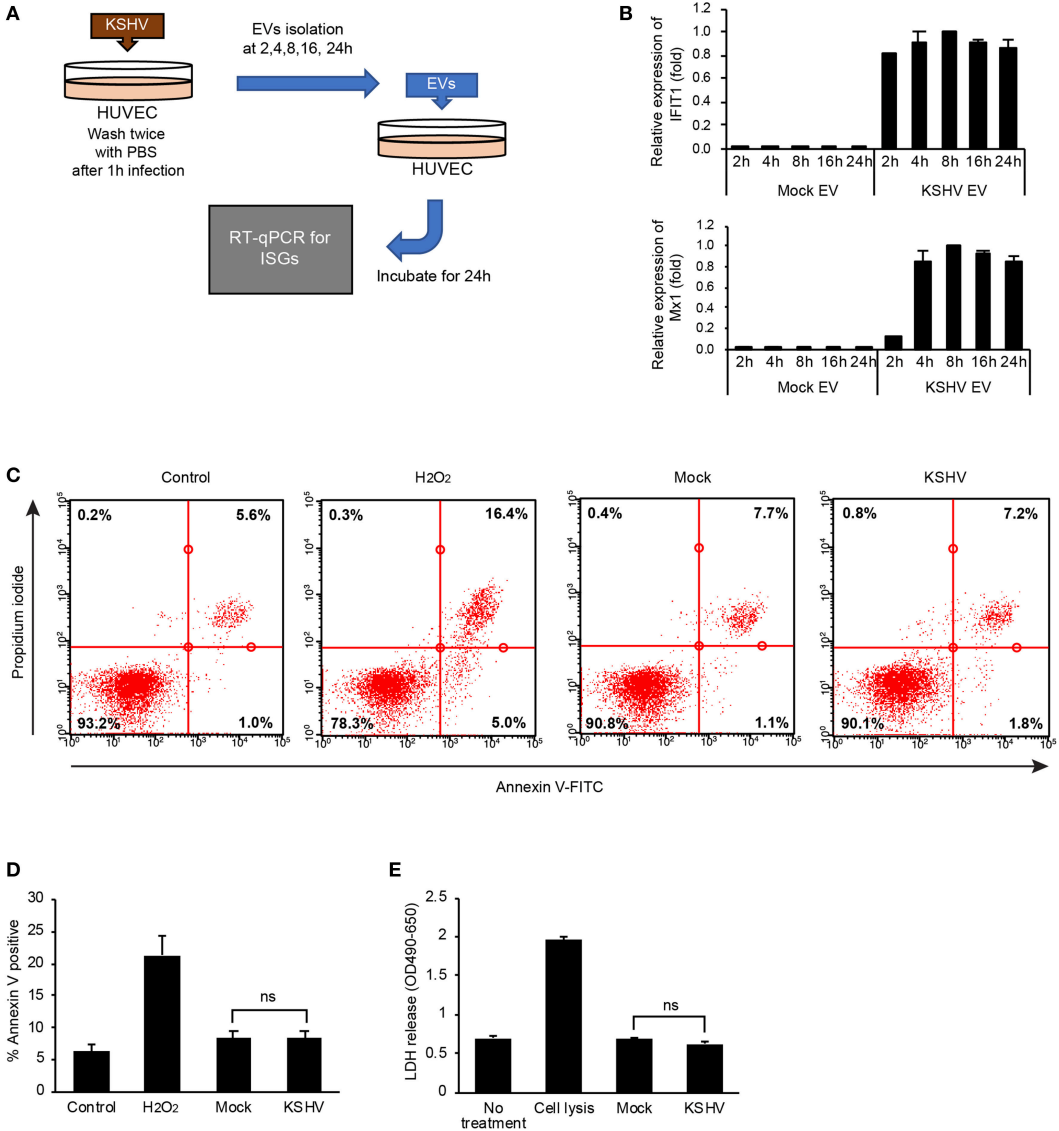
Stimulation of ISG expression by KSHV EVs is not associated with a production of virus or cell death. **(A)** Schematic summary of the experimental process. KSHV infected to HUVECs for indicated periods and EVs were isolated from the supernatant of KSHV-infected cells. Then, each isolated EVs was treated with HUVECs for 24 h, and mRNA expressions for ISGs were analyzed. **(B)** mRNA expression of mock EVs vs. KSHV EVs-treated HUVECs. Each time point represents the time that EVs were isolated after KSHV infection. Data are shown as the mean ± SD, *n* = 6. **(C,D)** Apoptosis and cell death in KSHV-infected HUVECs at 8 h of postinfection. Mock- or KSHV-infected HUVECs were detached from culture plate and stained with FITC-conjugated Annexin V and propidium iodide. Representative and average values from three independent experiments are shown in **(C)** and **(D)**, respectively. PBS and H_2_O_2_ were used as negative and positive control, respectively. Data are shown as the mean ± SD, *n* = 3. ns, not significant. **(E)** LDH assay for mock- or KSHV-infected HUVECs at 8 h of postinfection. Data are shown as the mean ± SD, *n* = 6. ns, not significant.

In our initial study design, we isolated KSHV EVs at 24 h of postinfection and treated them with HUVECs. To determine the time taken to release an effective EV for ISGs after KSHV infection, KSHV EVs were isolated at various time points after KSHV infection (Figures 3A,B). Interestingly, 4 h after KSHV infection was enough for the isolated KSHV EVs to induce ISG expression, confirming that the induction of ISGs would not be associated with KSHV because KSHV is generally produced 48 h of postinfection (16). To confirm the presence of viral nucleic acids and proteins, KSHV ORF26 was amplified from KSHV EVs by PCR, and KSHV envelope protein, ORF65, was analyzed by western blotting (Supplementary Figure 2A). We could not detect KSHV DNA or viral protein in KSHV EVs. Furthermore, viral particles or viral gene expressions in KSHV-infected or KSHV EVs-treated cells were also investigated. As expected, viral particles or viral gene expressions were not detected in KSHV EVs-treated HUVECs (Supplementary Figures 2B–E). These results showed that KSHV EVs did not contain KSHV virion, suggesting KSHV EVs alone can cause ISG response without the virus. Some previous studies showed that EVs from apoptotic cells could induce inflammation by their harboring proteins or nucleic acids. For example, apoptotic bodies from endothelial cells contained IL-1α (17) and EVs from apoptotic T cell blasts triggered the secretion of IFN-α in plasmacytoid dendritic cells (18). Since ISGs might be stimulated by apoptosis or cell death, apoptosis and cell death in KSHV-infected HUVECs was analyzed at 8 h of postinfection, which was the highest time point of ISG expression. We could not find significantly increased apoptosis or cell death by KSHV infection in Annexin V/PI staining (Figures 3C,D). LDH release in the culture supernatant was also analyzed at the same time point (Figure 3E). In KSHV-infected cells, LDH release was not increased at all compared to mock-infected cells (Figure 3E). Taken together, our results indicated that the induction of ISGs by KSHV EVs would not be caused by a product from apoptosis or cell death during KSHV infection.

### Entry of KSHV EVs Is More Prominent Than Mock EVs

**Figure 4:**
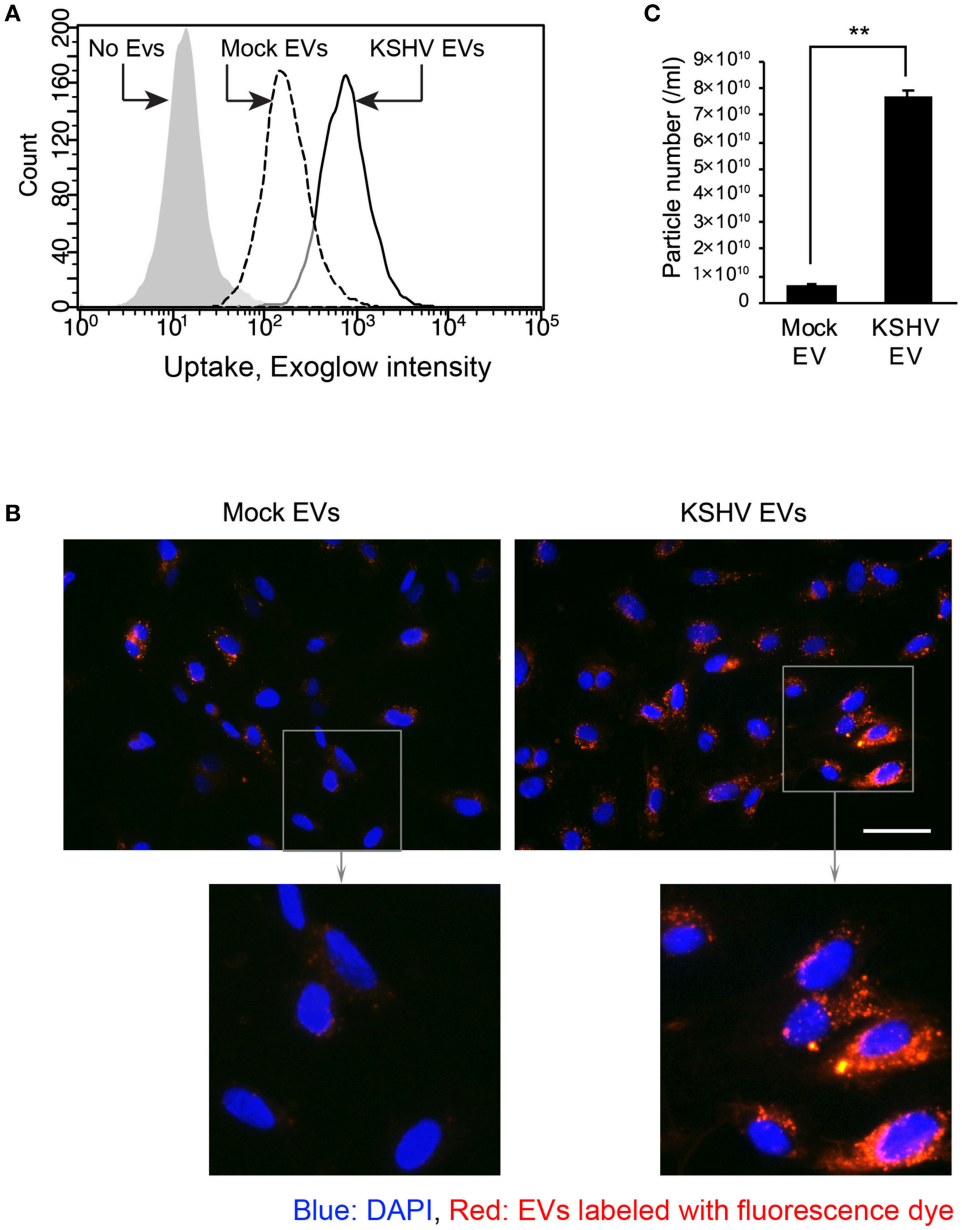
Entry of KSHV EVs into human endothelial cells. Mock EVs or KSHV EVs were isolated from the same amount of conditioned media, and each EV was labeled with fluorescence dye. The labeled EVs were treated with HUVECs, and their entry was analyzed by flow cytometry **(A)** and fluorescence microscopy **(B)**. Scale bar: 50 μm. **(C)** Particle number of the EVs of experiments-applied. Data are shown as the mean ± SD, *n* = 3, ^**^p < 0.01.

To investigate whether KSHV EVs were taken up by HUVECs, EVs were stained with a fluorescence dye, Exoglow. Then, the labeled EVs were treated to HUVECs, followed by analyzing their entry by flow cytometry and fluorescent microscopy (Figures 4A,B). Compared to mock EVs, KSHV EVs-treated cells showed higher fluorescence intensity in flow cytometry (Figure 4A). More particles of EVs were also detected in the microscopic analysis (Figure 4B). In nanoparticle tracking analysis, an overall 10-fold higher number of particles was detected in EVs from KSHV-infected cells than those from mock-infected cells (Figure 4C). Therefore, increased entry of EVs in KSHV EV-treated cells may be caused by the larger quantity of EVs.

### Induction of ISGs Is Specifically Mediated by KSHV EVs

**Figure 5:**
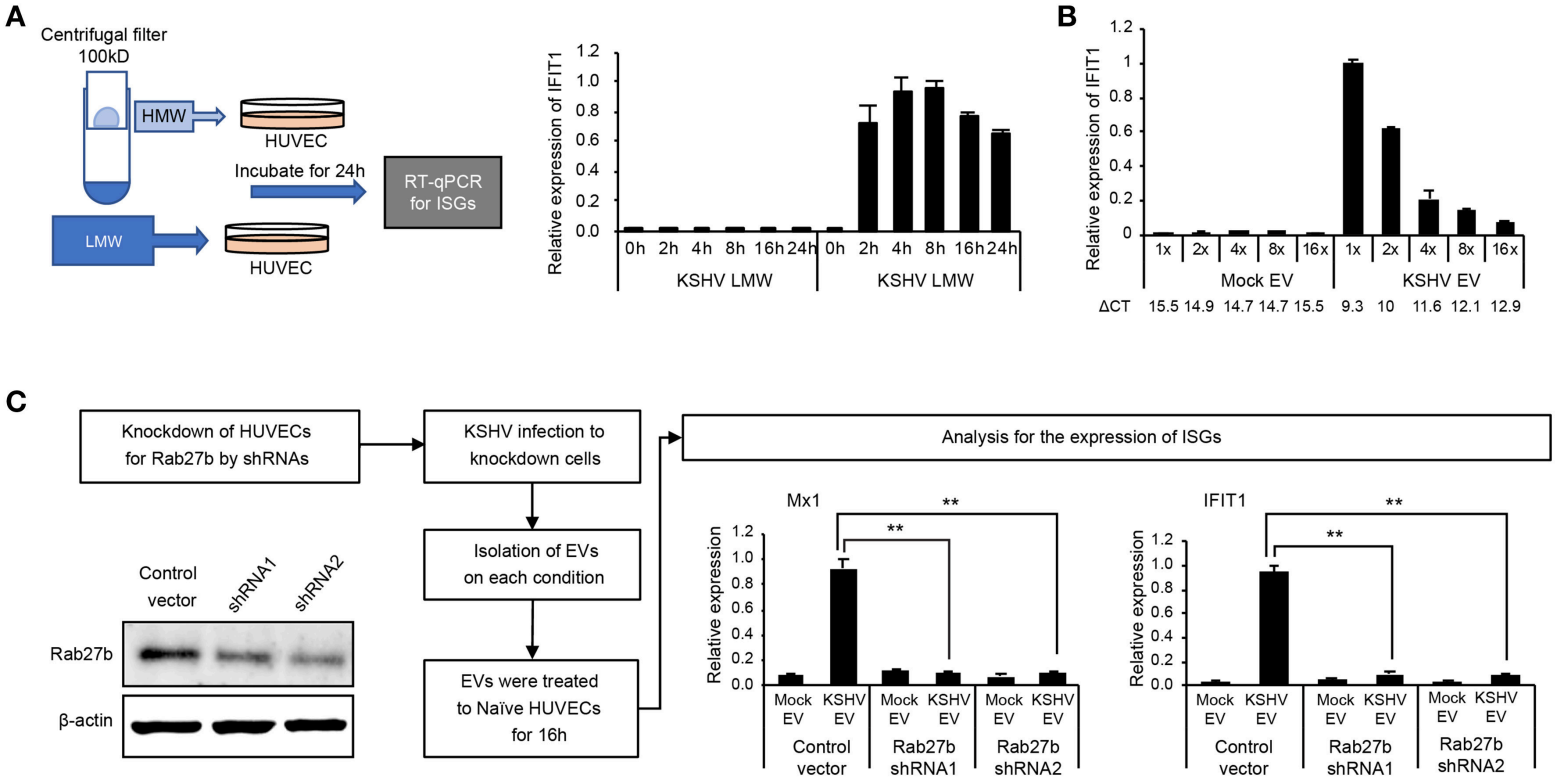
EV was an essential factor in the induction of ISGs by the supernatant from KSHV-infected cells. **(A)** KSHV EVs isolated by centrifugal filtration induced IFIT1 expression. The supernatant from KSHV-infected cells was separated by centrifugal filter device with a cut-off of 100 kDa. High molecular weight (HMW) proteins (the retained materials by a filter) and low molecular weight (LMW) proteins (the flow-through) was applied to HUVECs, followed by analyzing mRNA expression by RT-qPCR. **(B)** Induction of ISGs was correlated with the amount of EVs. The same volume of EVs was isolated from the same amount of the supernatant from mock- or KSHV-infected cells. Then, each EV was applied to HUVECs, followed by analyzing mRNA expression by RT-qPCR. ΔCT indicated the normalized CT value of IFIT1 with reference gene, β-actin. **(C)** Knockdown of Rab27b suppressed the induction of ISGs by KSHV EVs. The expression of Rab27b was suppressed by shRNA in HUVECs. After KSHV infection, mock EVs or KSHV EVs were isolated from the Rab27b-suppressed HUVECs. Each prepared EV was applied to uninfected HUVECs, and mRNA expressions for ISGs were analyzed. The grouping of blots cropped from different gels and full-length blots are included in a Supplementary Figure 5. Data are shown as the mean ± SD, *n* = 6, ^**^p < 0.01.

To confirm whether KSHV EVs-mediated ISGs response is not stimulated by cytokines or small proteins from KSHV-infected cells, the conditioned medium containing KSHV-infected cells was separated into high molecular weight (HMW) proteins and low molecular weight (LMW) proteins using centrifugal filter device, Amicon ultra-2 100 kDa. Each of them added to uninfected HUVECs, and the mRNA expression of IFIT1, a representative ISG, was analyzed (Figure 5A). While KSHV HMW proteins-treated cells showed highly upregulated IFIT1, KSHV LMW proteins did not induce its expression. These results are consistent with the previous results with EVs isolated by differential centrifugation (Figures 1, 2), suggesting that KSHV EVs-mediated ISGs expression would not be mediated by small-sized proteins including cytokines but by EVs or large-sized proteins. To investigate whether KSHV EV-mediated induction of ISGs depended on the amount of EVs, serially diluted EVs were treated with HUVECs. A dose-dependent decrease of IFIT1 expression was observed in KSHV EV-treated cells (Figure 5B), indicating that our results meet the requirements of dose-response studies of EVs recommended in MISEV2018 (19). Furthermore, 16-fold diluted KSHV EVs induced higher expression of IFIT1 in HUVECs compared to 1-fold diluted mock EVs, suggesting a similar number of KSHV EVs still induce the expression of ISGs compared to mock EVs. Next, to confirm if induction of ISGs was specifically mediated by EVs, an essential protein for biogenesis of EVs, Rab27b, was suppressed by shRNA in HUVECs (Figure 5C). After 2 weeks of incubation with a selection marker, puromycin, for the shRNA-transduced cells, the expression of Rab27b decreased in knockdown cells. The prepared cells were infected with KSHV, and EVs were isolated from each culture supernatant. These isolated EVs were treated to uninfected HUVECs, followed by analysis of mRNA expression of MX1 and IFIT1. mRNA expressions of both ISGs were significantly suppressed in HUVECs treated with KSHV EVs from Rab27b knockdown cells, indicating biogenesis of EVs as a critical factor for KSHV EVs to induce ISGs in human endothelial cells.

### Mitochondrial DNA on KSHV EVs Originated From the Cytosol of KSHV-Infected Cells Is a Stimulant for ISGs

**Figure 6:**
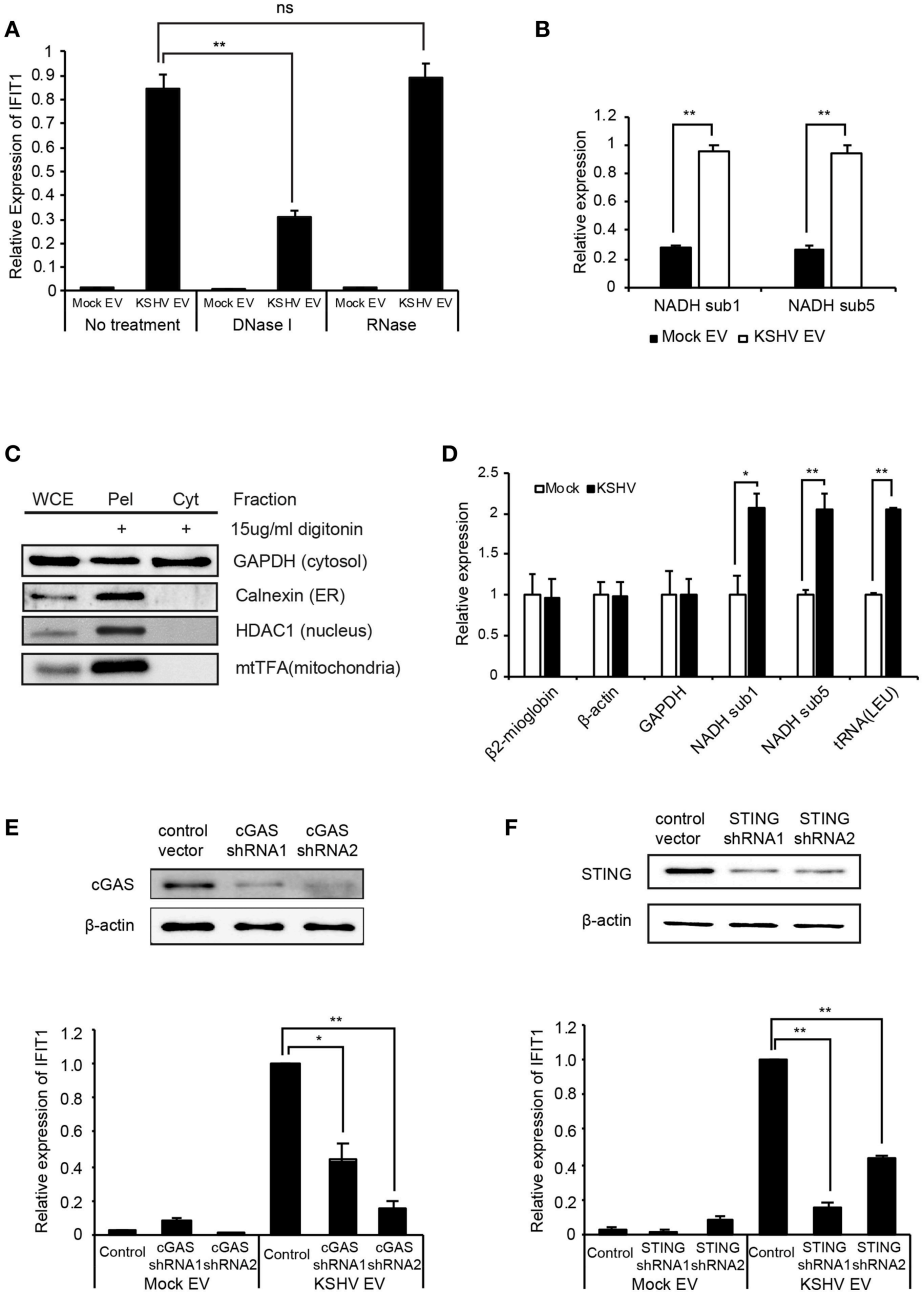
Induction of ISGs by KSHV EVs is associated with mtDNA. **(A)** mRNA expression of IFIT1 in HUVECs treated with DNase I or RNase-treated EVs. **(B)** Quantification of mtDNA in mock EVs vs. KSHV EVs. Genomic DNA was isolated from the same number of EVs, and mtDNA-related genes were analyzed by qPCR. **(C)** Western blotting for the cytoplasmic fraction from KSHV-infected HUVECs. Cytoplasmic fraction was extracted by digitonin, and its purity was analyzed by western blot analysis. WCE: whole cell extract, Pel: pellet after extraction of the cytoplasmic fraction, Cyt: cytoplasmic fraction. **(D)** Quantification for genomic and mitochondrial DNA in the cytoplasmic fraction from mock- vs. KSHV-infected HUVECs. **(E,F)** Induction of IFIT1 in cGAS or STING-suppressed HUVECs by KSHV EVs. The expression of cGAS **(E)** or STING **(F)** was suppressed by shRNAs. Mock EVs or KSHV EVs were treated with each indicated knockdown cell, and the induction in IFIT1 expression was analyzed by RT-qPCR. Data are shown as the mean ± SD, *n* = 6, ns, not significant, ^*^p < 0.05, and ^**^p < 0.01.The grouping of blots cropped from different gels and full-length blots are included in a Supplementary Figures 6–8.

Nucleic acids are recognized by the innate immune system, which provides key signals to initiate antiviral responses, including ISGs (20, 21). To determine whether the DNA or RNA on EVs is associated with the induction of ISGs, the isolated EVs were treated with DNase I or RNase, followed by addition to HUVECs. Interestingly, only DNase I treatment of EVs significantly suppressed the expression of ISGs of HUVECs, as observed from the mRNA expression data (Figure 6A). These results indicate that the external DNA on EVs might be one of the causative factors for the induction of ISGs, which is consistent with recent studies showing that the external dsDNA on EVs could be an inducing agent for inflammation (22, 23). A previous study showed that mitochondrial DNA (mtDNA) stress primed the antiviral innate immune response (24, 25). Moreover, Sun et al. indicated that infection of dengue virus activates innate immune response via the release of mtDNA (26). Therefore, we analyzed mtDNA in EVs from mock and KSHV-infected cells (Figure 6B). Interestingly, a larger quantity of mtDNA was detected in KSHV EVs than in mock EVs, which is consistent with DNase I-treated experiments (Figure 6A). We also found that the quantity of mtDNA of EVs was increased in the time course of KSHV infection (Supplementary Figure 3). For genomic DNA in the same samples, we could not find an amplification of GAPDH and β-actin (data not shown). To determine whether KSHV-infected HUVECs release mtDNA into the cytosol, we extracted the cytosolic fraction from KSHV-infected cells without any contamination of the nucleus or other cellular organelles (Figure 6C). Genomic and mitochondrial DNA were analyzed in the cytosolic fraction derived from mock- or KSHV-infected cells (Figure 6D). The cytosolic fraction from KSHV-infected cells contained a larger amount of mtDNA than that from mock-infected cells, which might be the origin of mtDNA of KSHV EVs. We next examined the involvement of the cytosolic DNA sensor cGAS in mtDNA stress signaling, as it mediates ISG expression in response to exogenous and endogenous immunostimulatory DNA species. Knockdown of cGAS in KSHV EVs-treated HUVECs significantly suppressed IFIT1 expression (Figure 6E). Besides, IFIT1 mRNA in KSHV EVs-treated HUVECs were also reduced upon STING knockdown (Figure 6F), indicating that cGAS-STING signaling would be a driver of KSHV EVs-induced ISG expression. STING signals via the TBK1-IRF3/7 axis to trigger antiviral gene expression. In the microarray analysis (Figures 1C,D), TBK1 was analyzed as the top regulator of effect network in KSHV EVs-treated HUVECs, which supports the association of cGAS-STING pathway in KSHV EVs-treated cells. Taken together, these results indicate that mtDNA from KSHV EVs facilitates cGAS-dependent sensing of cytoplasmic mtDNA, resulting in STING-TBK-IRF3 signaling to trigger ISG expression.

### Antiviral Effect of KSHV EVs in Human Endothelial Cells

**Figure 7:**
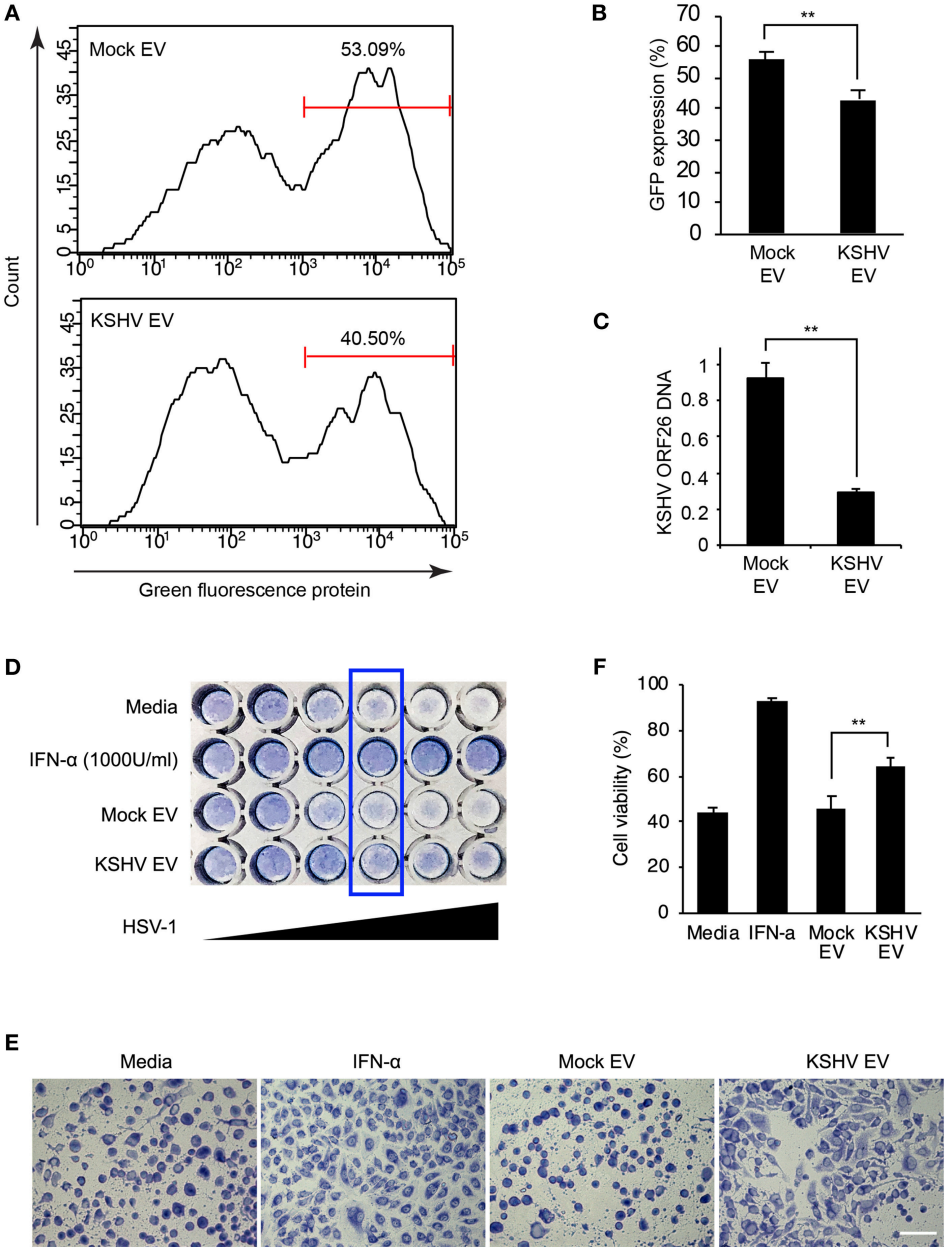
Antiviral effect of KSHV EV in human endothelial cells. **(A–C)** KSHV infectivity was decreased by KSHV EVs. Mock EVs and KSHV EVs were pretreated to human endothelial cells for 24 h, and KSHV infected into the prepared cells. KSHV infectivity was analyzed by flow cytometry through GFP expression **(A,B)**. After KSHV infection, KSHV DNA was compared between mock EVs vs. KSHV EVs-treated cells **(C)**. Data are shown as the mean ± SD, *n* = 3, ^**^p < 0.01. **(D–F)** KSHV EVs inhibit the infection of HSV-1. HSV-1 (MOI = 64) was serially diluted into mock EVs or KSHV EVs-treated cells. After 24 h of incubation, the cytopathic effect was analyzed by staining (D). Cellular morphology in the wells of boxed area from **(D)** was visualized by microscopy **(E)**. Scale bar: 100 μm. **(F)** The ratio of cell viability was measured by the WST-1 assay (MOI of HSV-1 = 4). Data are shown as the mean ± SD, *n* = 4, ^**^p < 0.01.

To establish a functional significance of KSHV EVs-induced antiviral priming, KSHV was challenged with the KSHV EVs-pretreated HUVECs. In contrast to mock EVs-treated HUVECs, KSHV EVs-treated cells showed significantly less infectivity for KSHV (Figures 7A,B). From those cells, genomic DNA was isolated and KSHV ORF26 DNA was quantified by real-time PCR (Figure 7C). KSHV EVs-treated cells showed significantly lesser KSHV DNA than mock EVs-treated cells, which is consistent with KSHV infectivity results. To evaluate the antiviral effect against another virus, human herpes simplex virus type 1 (HSV-1) was used to infect EVs-pretreated HUVECs. For HSV-1 infection, more live cells were observed in KSHV EVs-treated cells than in mock EVs-treated cells (Figures 7D–F), indicating that KSHV EVs provide higher resistance to HSV-1 infection in HUVECs than mock EVs.

## Discussion

To protect multicellular organisms against viruses, it is vital that infected cells trigger antiviral defense responses that can be rapidly transmitted to non-infected cells. The spread of innate immune responses is generally attributed to the production of cytokines, including type I IFNs, which have broad antiviral activities through the induction of ISGs (6).

Increasing evidence suggests that EVs from some virus-infected cells modulate cellular processes including immune responses (27–29). Studying EVs in viral infections poses a limitation: separation of EVs from viral particles is challenging. In our previous study, EVs were successfully isolated from KSHV-infected cells in the early phase of infection (15). Using these EVs from KSHV-infected cells, we demonstrated that EVs from KSHV-infected cells trigger an antiviral response by inducing ISGs in human endothelial cells. There have been a few studies on EVs from KSHV-infected cells (30, 31). However, mostly viral microRNA in EVs have been highlighted so far. In this study, we showed that KSHV EVs stimulate ISGs in bystander cells using host mtDNA, demonstrating that virus-infected cells can mediate early antiviral defenses by modulating the production and content of EVs. An EVs-mediated antiviral effect may provide the basis for therapeutic strategies to control viral infection.

In hepatitis B and C viral infections, an antiviral effect could be transferred from cell to cell through exosomes (4, 32). These studies showed that EVs could deliver not only viral components but also molecules with antiviral activity. To our best knowledge, this is the first study that demonstrates EVs with mtDNA from virus-infected cells to be a triggering factor for an antiviral response. Previous studies showed that mtDNA activates innate immune responses through cGAS or TLR9 (24, 33, 34). Additionally, cGAS-mediated antiviral signaling was spread from dengue virus-infected cells to neighboring cells via gap junctions using mtDNA (26). Considering all of these observations, the antiviral response by EVs containing mtDNA seems to be a reasonable response to viral infection. EVs mediate intercellular communication and regulate immune signaling. Previous studies indicated that double-stranded genomic DNA is located in circulating EVs and a large proportion of human blood plasma cell-free DNA is localized in EVs (35, 36), suggesting that blood circulating DNA or anti-DNA antibodies in autoimmune diseases might be associated with DNA-containing EVs.

In this study, we demonstrated that KSHV-infected cells release approximately 10-folds of EVs particles compared to uninfected cells, which might be associated with extruding the increased mtDNA in the cytosol of KSHV-infected cells to favor cell survival. Cytosolic mtDNA accumulates have known to trigger cell injury (37). In patients with non-alcoholic steatohepatitis, hepatocytes have shown to release mtDNA through microparticles (38). Therefore, the secretion of mtDNA through EVs might be a mechanism for cellular homeostasis. Although the exact functions and mechanisms remain to be elucidated, some virus-infected cells showed increased production of EVs (15, 39, 40). As the small Rab GTPase are well-known to control the secretion of EVs (41, 42), some Rab proteins appear to be factors to regulate the release of EVs in virus-infected cells. Infection of CMV increased the level of Rab27a, which was related to CMV production (43). HSV-1 also exploits Rab27a for its intracellular transport and exocytosis (44, 45). Interaction of virus and Rab GTPase might modulate not only the production of the virus but also the release of EVs. Another pathway that might lead to EVs production in virus-infected cells is the tetraspanin-dependent pathways (46). A recent paper showed that HSV-1 triggered the release of CD63 positive EVs but not alter the exocytosis of TSG101 or Alix, suggesting the infection triggers ESCRT-independent pathways for the release of EVs (47). Understanding and manipulation of EVs biogenesis during virus infection may reveal potential targets for antiviral therapy.

While we suggest mtDNA is a causative factor for the stimulation of ISGs by KSHV EVs, the mechanisms of KSHV EVs-mediated ISGs response is not entirely resolved here, and other factors and pathways may be associated with them. We could not extensively investigate the effect of DNA inside the EVs because DNase treatment removed only surface DNA and permeabilizing agent disrupted a functional structure of EVs. More research should be required to elucidate the exact mechanisms of EVs-mediated antiviral response and their biological significance *in vivo*. Nevertheless, we provide clear evidence that EVs from KSHV-infected HUVECs restricted infection of KSHV and HSV-1, suggesting that DNA-carrying EVs might be important mediators for antiviral response. Taken together, our findings would contribute to the current understanding of the antiviral immune response of EVs from virus-infected cells.

## Author Contributions

M-SL designed the study. HJ, JL, SL, S-KK, SP, S-MY, and M-SL performed the experiments and analyzed data. HJ and M-SL wrote the manuscript. All authors read and approved the final manuscript.

### Conflict of Interest Statement

The authors declare that the research was conducted in the absence of any commercial or financial relationships that could be construed as a potential conflict of interest.
