# Supplementary material for: Extracellular Vesicles From KSHV-Infected Cells Stimulate Antiviral Immune Response Through Mitochondrial DNA
Source: Front Immunol. 2019 Apr 24;10:876. doi: 10.3389/fimmu.2019.00876 (PMC6491682; doi:10.3389/fimmu.2019.00876)
Supplement: Supplementary file 1 [file Data_Sheet_1.docx]

Supplementary Material


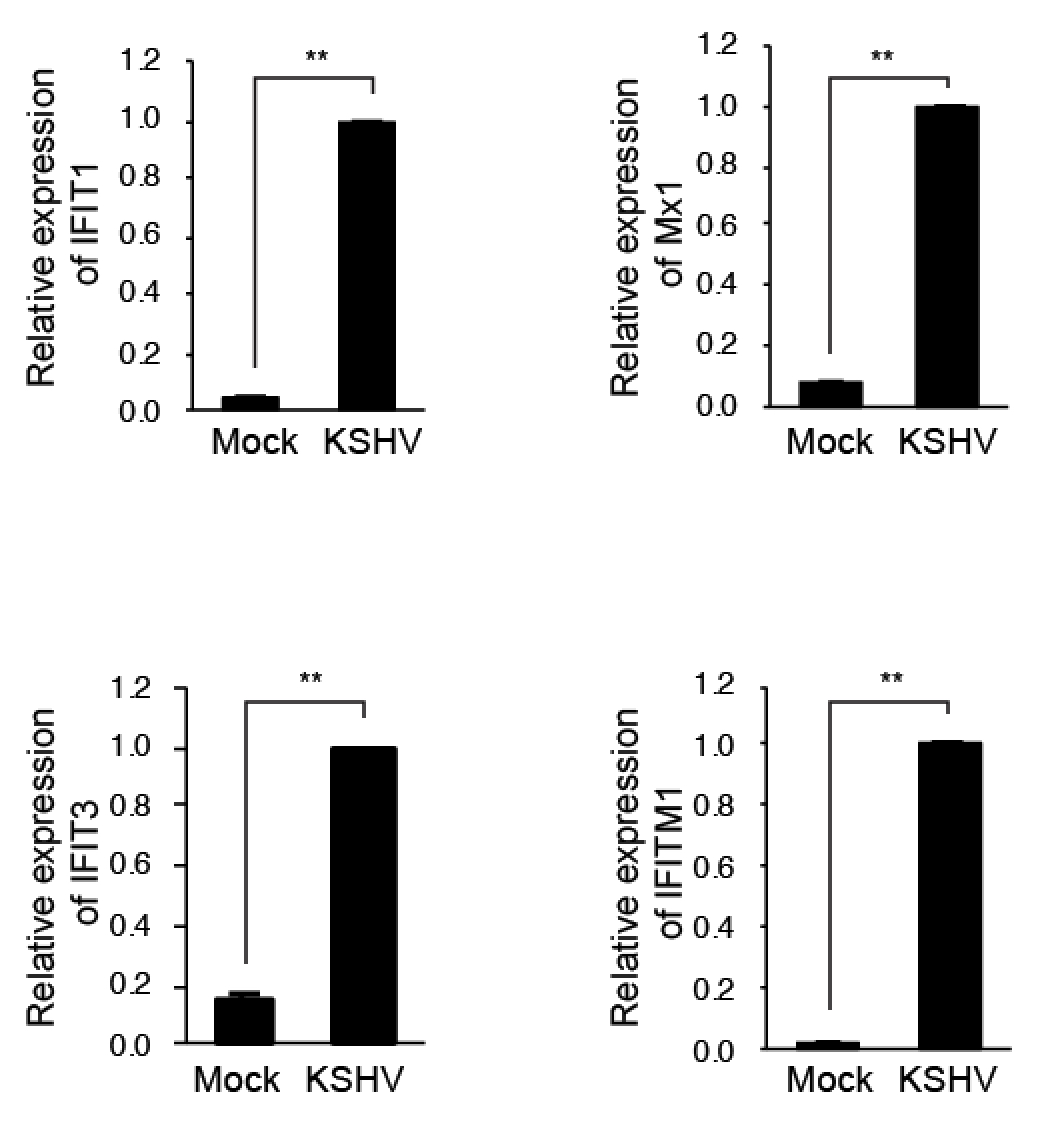


**Supplementary Figure 1.** Increased expression of ISGs in human endothelial cells upon KSHV infection. After KSHV infection, RNA was isolated at 24 h of postinfection, and mRNA expression of the indicated ISGs was analyzed by RT-qPCR. Data are shown as the mean ± SD, n = 6, **p < 0.01.


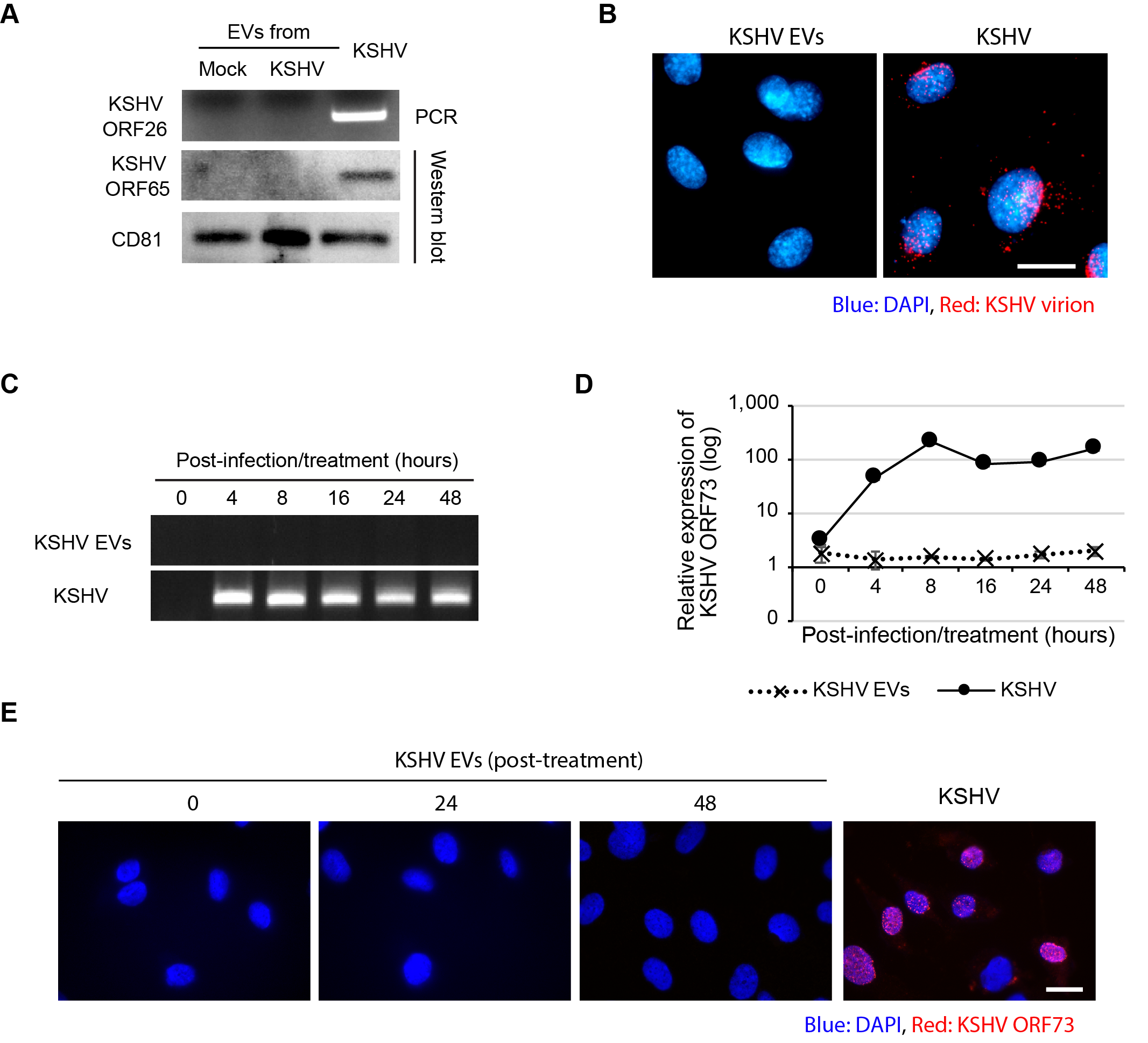


**Supplementary Figure 2. The absence of KSHV in EVs from KSHV-infected cells.** (A) Detection of KSHV viral genome and protein in EVs from KSHV-infected cells. Genomic DNA or protein was extracted from the same quantity of EVs or KSHV (5 μg each). KSHV ORF65 or CD81 was detected by western blot analysis. KSHV genome was investigated by PCR reaction using the specific primers for KSHV ORF26. (B) Detection of viral particles in KSHV EVs-treated cells. HUVECs were treated with EVs from KSHV-infected cells or infected with KSHV for 4 hours, and they were stained for KSHV particles by IFA using a monoclonal antibody to KSHV ORF65. Nuclei were stained with DAPI. Scale bar: 20 μm. (C-D) KSHV ORF73 mRNA expression in KSHV EVs-treated cells. After treatment of KSHV EVs or infection of KSHV, mRNA expression of KSHV ORF73 was analyzed by quantitative RT-PCR. The results were presented by agarose gel electrophoresis (C) or relative expression in qPCR. (E) The expression of KSHV ORF73 after KSHV EVs-treated HUVECs. After treatment of KSHV EVs or infection of KSHV, KSHV ORF73 was detected by IFA. Scale bar: 20 μm.


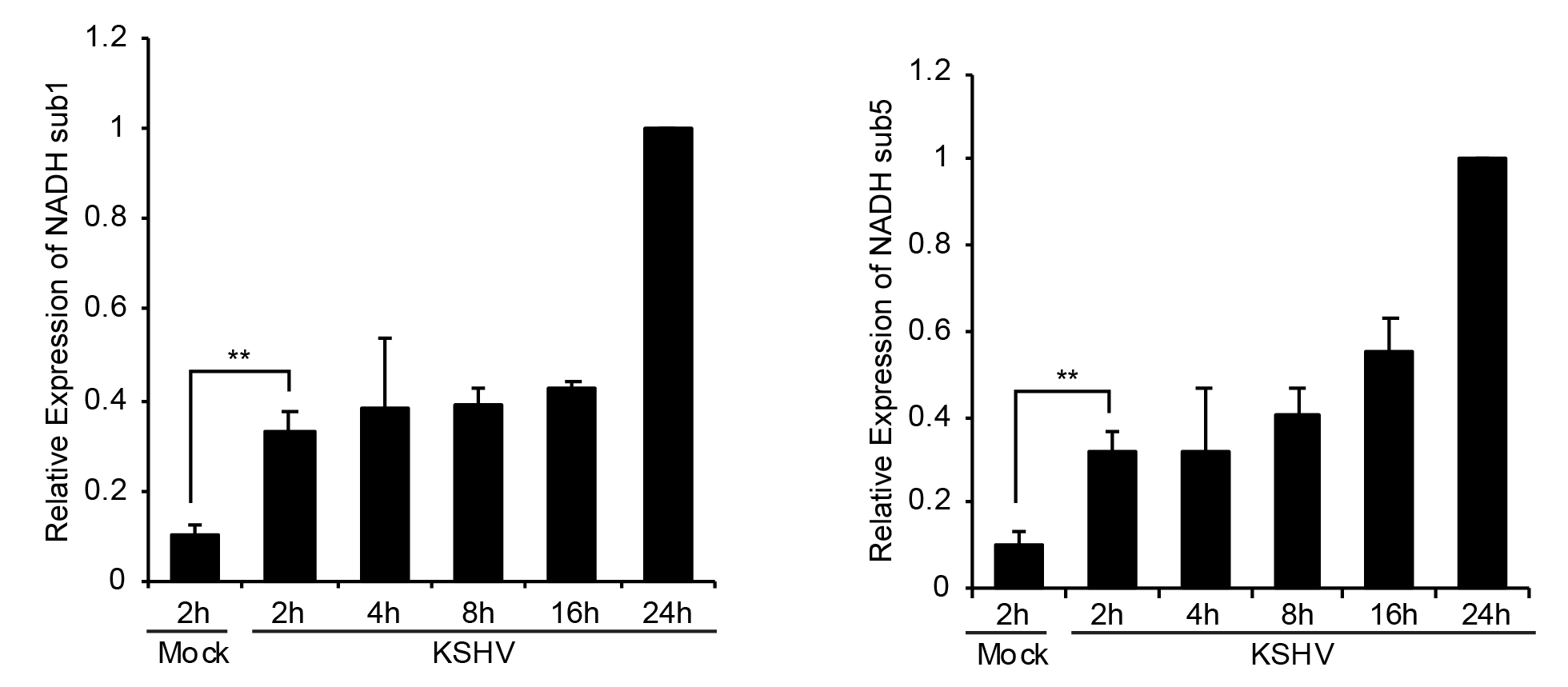


**Supplementary Figure 3. The increase of mtDNA in KSHV EVs after KSHV infection.** After KSHV infection, EVs were isolated at each indicated time points. The quantity of mtDNA in the isolated EVs was analyzed by qPCR. Mock EVs at 2 h was used as a control. Data are shown as the mean ± SD, n = 6, **p < 0.01.


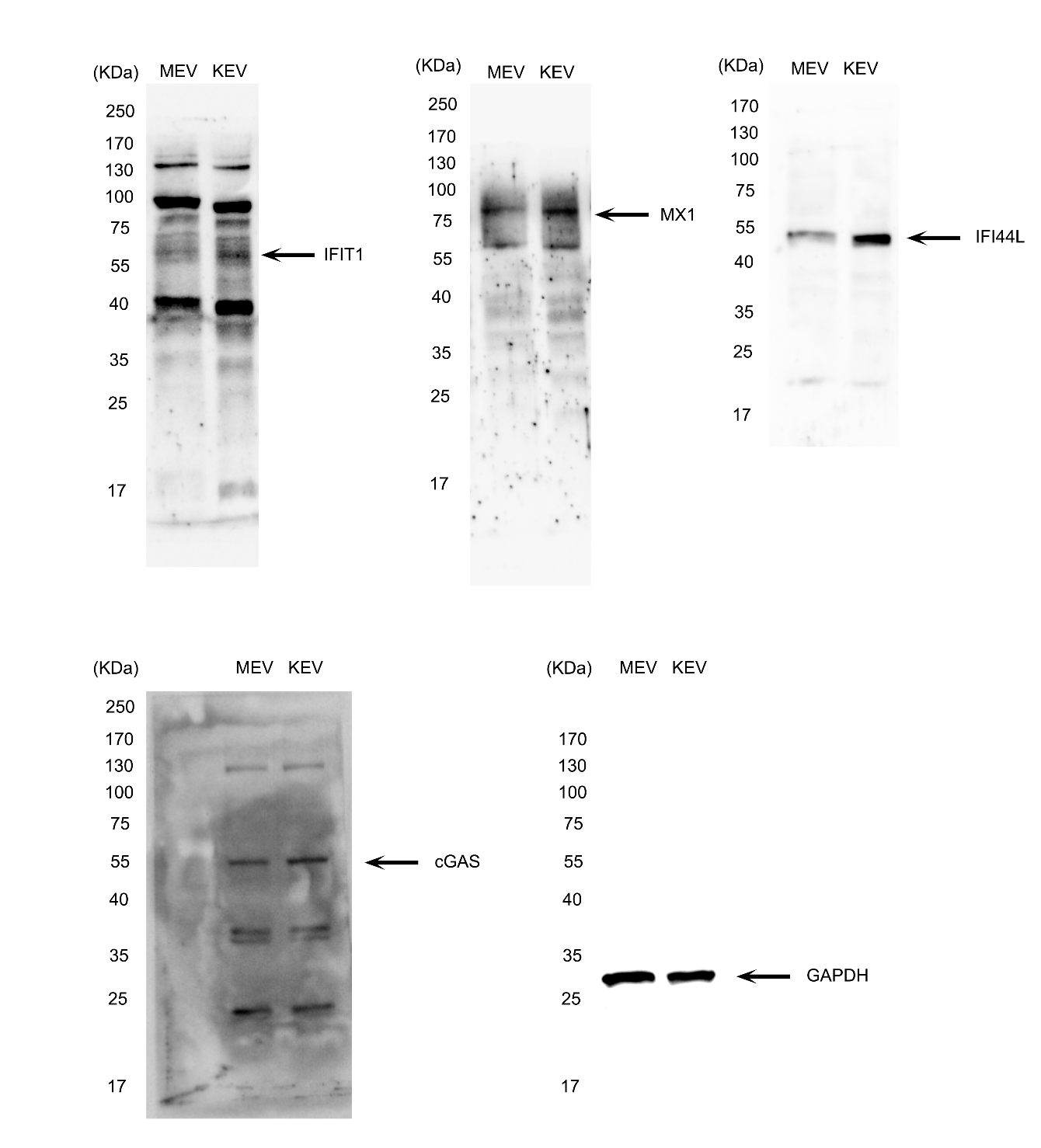


**Supplementary Figure 4.** Western blot uncut membrane data of Figure 2B.


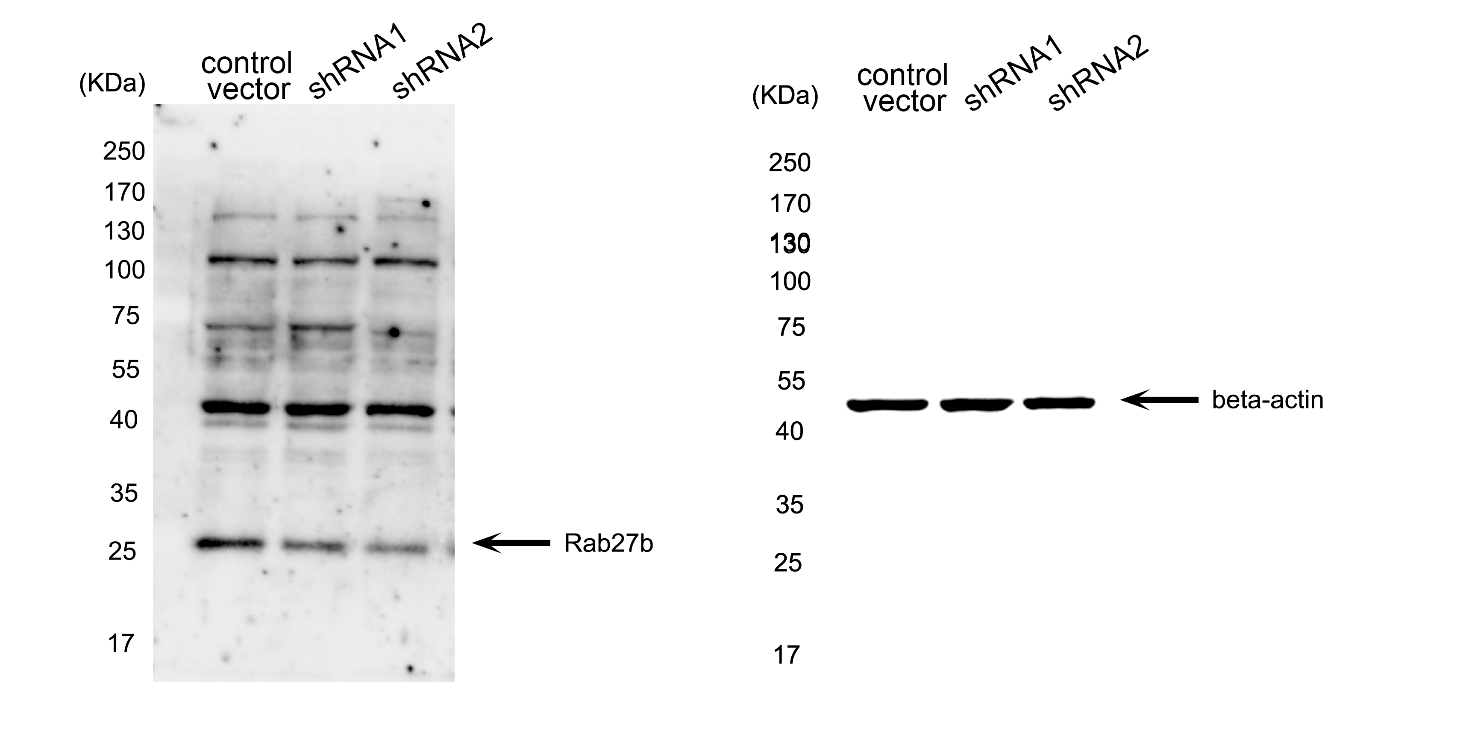


**Supplementary Figure 5.** Western blot uncut membrane data of Figure 5C.





**Supplementary Figure 6.** Western blot uncut membrane data of Figure 6C.


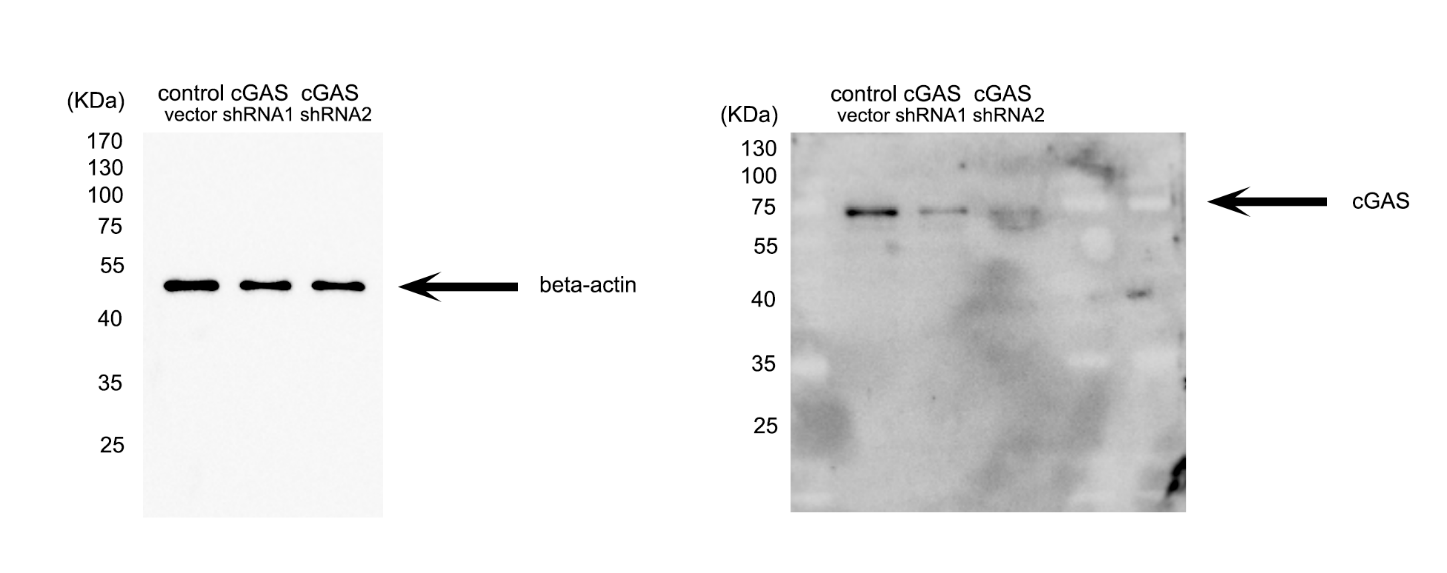


**Supplementary Figure 7.** Western blot uncut membrane data of Figure 6E.


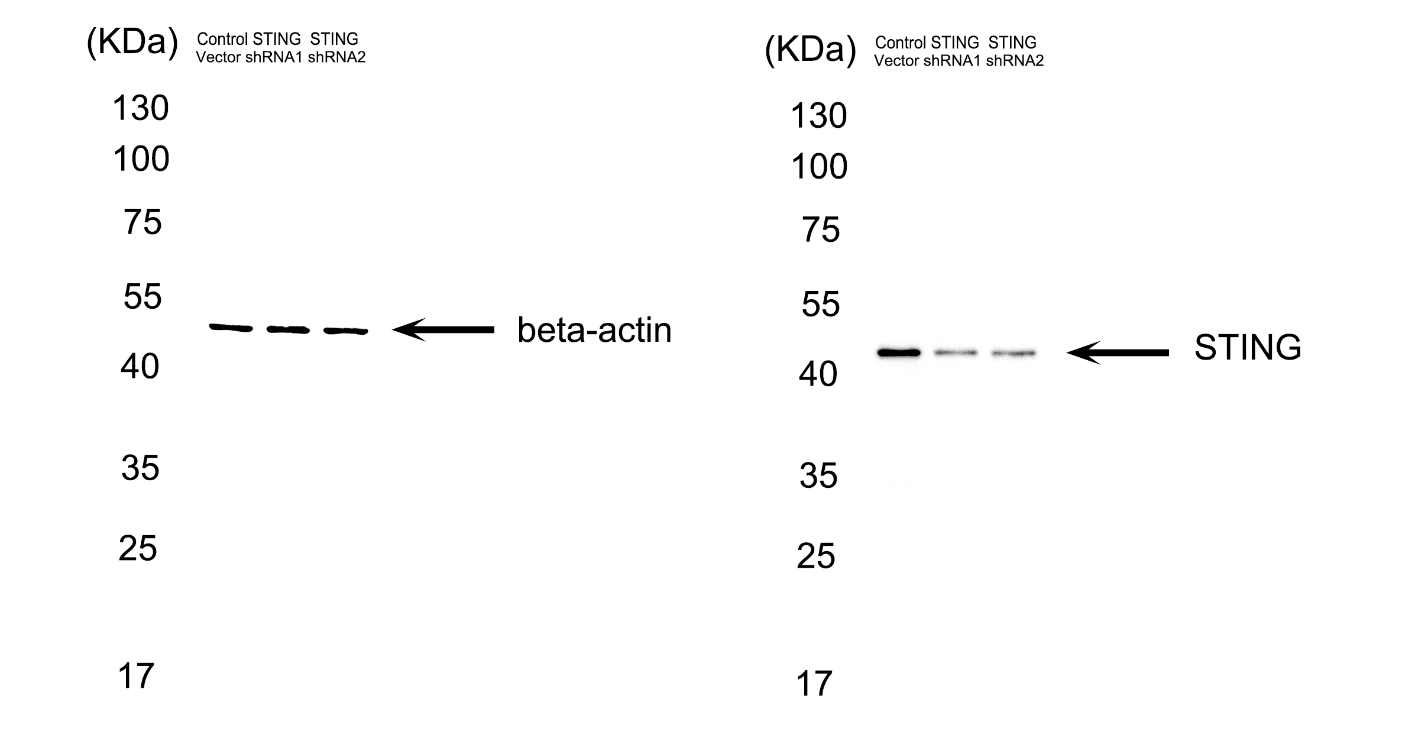


**Supplementary Figure 8.** Western blot uncut membrane data of Figure 6F.
